# Influence of the Degumming Process Parameters on the Formation of Glyceryl Esters and 3-MCPDE in Refined Palm Oil: Optimization and Palm Oil Quality Analyses

**DOI:** 10.3390/foods11010124

**Published:** 2022-01-05

**Authors:** Mohammad Saiful Nidzam, Md. Sohrab Hossain, Norli Ismail, Razam Abdul Latip, Muhammad Khalish Mohammad Ilias, Md. Bazlul Mobin Siddique, Muzafar Zulkifli

**Affiliations:** 1 School of Industrial Technology, Universiti Sains Malaysia (USM), Gelugor 11800, Malaysia; saiful.nidzam.ismail@simedarbyplantation.com (M.S.N.); norlii@usm.my (N.I.); muhdkhalish.ilias@student.usm.my (M.K.M.I.); 2 Sime Darby Plantation Research Sdn Bhd, Pulau Carey, Kuala Langat, Pulau Carey 42960, Malaysia; razam.latip@simedarbyplantation.com; 3 Faculty of Engineering, Computing and Science, Swinburne University of Technology, Kuching 93350, Malaysia; msiddique@swinburne.edu.my; 4 Green Chemistry and Sustainability Cluster, Branch Campus, Malaysian Institute of Chemical and Bioengineering, Technology University Kuala Lumpur, Taboh Naning, Alor Gajah, Melaka 78000, Malaysia

**Keywords:** degumming, 3-MCPDE, glyceryl esters, palm oil refining, physical refining

## Abstract

The presence of glyceryl esters (GE) and 3-monochloropropane-1,2-diol esters (3-MCPDE) in refined, bleached, and deodorized (RBD) palm oil is severely concerning to the palm oil consumer. In the present study, the influence of the phosphoric acid degumming process on the formation of GE and 3-MCDE and in the RBD palm oil was determined with varying the acid dose (0.03–0.06 wt%), temperature (70–100 °C), and reaction time (15–45 min). The experimental conditions of the acid degumming process were designed following the central composite design of experiments, and they were optimized using Response Surface Methodology (RSM) based on the minimal formation of GE and 3-MCDE in the RBD palm oil. The optimal experimental conditions of the acid degumming process were a reaction time of 30 min, phosphoric acid concentration of 0.06 wt%, and temperature of 90 °C. Under these experimental conditions, the minimal GE and 3-MCDE formation in RBD palm oil were determined to be 0.61 mg/kg and 0.59 mg/kg; respectively. Several analytical methods were employed to determine RBD palm oil quality, including color, phosphorus, free fatty acids (FFAs), peroxide values, and fatty acid properties. It was found that the phosphoric acid degumming of CPO effectively removed the phosphorus and hydroperoxide content without conceding the quality of palm oil.

## 1. Introduction

Palm oil is the utmost consumed vegetable oil worldwide due to its lower production cost, nutrition values, and higher per hectare yield than any other oilseed crop [1,2]. Generally, the palm oil produced from oil palm fruits consists of palm kernel and mesocarp fiber. Palm oil’s most outstanding feature is the presence of a saturated and unsaturated fatty acid ratio of 1:1, which is a nutritionally favorable fatty acid content for human consumption [3]. Palm oil’s relatively high melting point enables it to have widespread application in the food industry. However, crude palm oil (CPO) cannot be consumed directly due to its containing undesirable substances, including free fatty acids (FFAs), gum, color, waxes, phosphate, toxic metal ion, and odoriferous substances [2,4]. Thus, there is a call for an effective refining process to remove palm oil’s undesirable impurities. There is also an increasing concern on GE and 3-MCPDE in refined palm oil. The European Food Safety Authority (EFSA) has categorized GE and 3-MCPDE in vegetable oil as genotoxic and carcinogenic, respectively [4]. However, both GE and 3-MCPDE are found as process contaminates, since these form in vegetable oil during the deodorization process due to the presence of triacylglycerol and diacylglycerol in degummed and bleached palm oil [5,6,7]. Both triacylglycerol and diacylglycerol present in degummed and bleached palm oil react with the chloride at high temperature of the deodorization process, resulting in producing GE and 3-MCPDE in refined palm oil [4,7]. Due to the genotoxic nature of GE, the EFSA in 2017 has set the limit of the presence of GE in vegetable oils is ≤1 mg/kg. Subsequently, the GE limit in palm oil has been followed by major food manufacturers companies, including Nestle and Unilever [3]. The 3-MCPD limits were set by EFSA to be at 2.5 ppm for palm oil and palm oil derivatives in 2018. However, the limits of 3-MCPDE in palm oil would be revised by consumers and are believed to be more stringent in the future [8].

The refining process of CPO could be conducted physically or chemically [9,10]. However, the physical refining process is preferable because of its substantial advantages over the chemical refining process, including higher oil yield, preventing excessive oil loss, minimizing the use of chemicals, and lowering the environmental impact of palm oil processing [2,8,11]. The physical refining process consists of three major processes: degumming, bleaching, and deodorization [8,11]. Degumming is the first step of the refining process of palm oil. The bleaching process is needed to extract the color pigments, protein degradation, traces of metal, and catalysts after the hydrogenation process [7]. The last unit process is the deodorization process. It is needed to eliminate the volatile components or the impurities that cause odor and off-flavor using steam distillation [8].

Although the formation of GE and 3-MCPDE in palm oil occurred during the deodorization process due to the high-temperature operation, an effective degumming process of the palm oil may minimize 3-MCPDE and GE formation during the deodorization process [8]. The prime goal of the degumming process is to eliminate gums or phospholipids from the oil. Usually, the crude palm oil extract from oil palm fruits contains phospholipid by about 0.5 to 2 wt% [9,12]. Without the degumming of the CPO, the phospholipids may present in the palm oil and interfere with the oil stability and undue darkening during the deodorization process [13]. Several types of degumming processes have been employed in the physical refining of palm oil, including water degumming, dry degumming, enzymatic degumming, acid degumming, membrane degumming, and EDTA degumming [12,13]. Among these degumming processes, acid degumming using a small amount of phosphoric acid (0.05 to 0.1 wt%) is the most utilized degumming process in the palm oil industry [7]. This is because phosphoric acid is an effective chelating agent, leading to the minimal amount of residual phospholipids in the oil [14]. It converts the nonhydratable gums into the hydratable form and, therefore, is easy to eliminate. In addition, the distinct characteristics of phosphoric acids as a degumming agent, such as chelating divalent metal ions, efficiency in removing phospholipids, and food grade, make it a preferable degumming agent in the palm oil refining process [8]. So far, there are a few studies available in the literature on the phosphoric acid degumming process and its impact on GE and 3-MCPDE formation in refined palm oil. Sim et al. [8] utilized the phosphoric acid degumming process in the physical refining of palm oil. The mitigation of the GE and 3-MCPDE formation obtained were 65% and 80%, respectively, at the optimal refining process of palm oil of 50 °C degumming temperature, 0.31% phosphoric dosage, 3% bleaching earth dosage, and 240 °C of deodorization temperature. Hew et al. [7] implemented the phosphoric degumming of palm oil to mitigate GE and 3-MCPDE formation in RBD palm oil. The study reported that the formation of GE and 3-MCPDE mitigated significantly at 0.06% of 85% phosphoric acid for 20 min. However, these studies reported that phosphoric acid doses, degumming temperature, and reaction time potentially influence the degumming of the palm oil and the mitigation of the GE and 3-MCPDE formation in RBD palm oil. However, there is limited study in the literature on the optimization of the degumming parameters such as phosphoric acid doses, degumming temperature, and reaction time on the mitigation of GE and 3-MCPDE formation in RBD palm oil.

Many variables may influence the degumming process, and therefore, it requires a quantitative assessment to determine the influence of the variables [15]. The conventional approach in the influence impact of process variable varies for one variable while other variables remain constant. This conventional approach may neglect the interactions between or among the variable studied in the degumming process. Generally, the design of experiments (DoE) is an effective statistical tool for designing experimental conditions, wherein the Response Surface Methodology (RSM) is an effective mathematical tool for optimizing the experimental conditions of a process. The potential feature of RSM is that it determines the influence of variables with minimal experimental trials. Therefore, studies have utilized the RSM to optimize the process variables and determine the interactions between or among the process variables [16]. In the present study, RSM was utilized to optimize the phosphoric acid degumming process on the minimal content of GE and 3-MCPDE formation in refined palm oil. The RBD palm oil properties such as free fatty acids (FFAs), color, phosphorous content, peroxide value, and fatty acids compositions were also determined to ensure the refined palm oil quality.

## 2. Materials and Methods

### 2.1. Materials

Crude palm oil was obtained from Sime Darby Oils Langat Refinery, Telok Panglima Garang, Selangor, Malaysia. The characterization of CPO is shown in Table 1. Phosphoric acid (purity 85%) was purchased from Nylex Chemical, Selangor, Malaysia. Acid-activated bleaching earth used during the bleaching process was obtained from Taiko Bleaching Earth Sdn. Bhd., Perak, Malaysia. The other chemicals utilized in the present study were in analytical grade.

### 2.2. Refining of CPO

**Figure 1:**
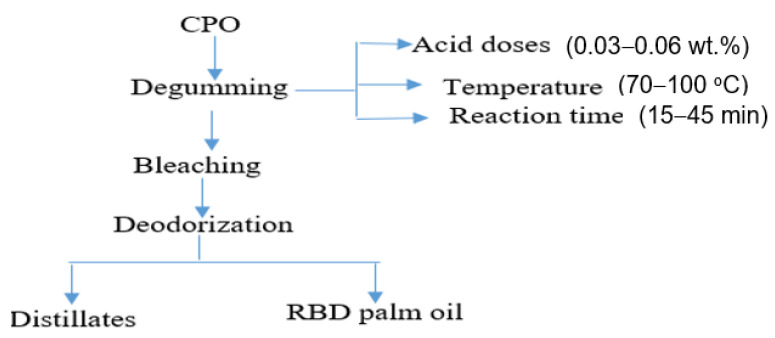
Physical refining process of crude palm oil.

The RBD palm oil was produced by the physical refining process of CPO. The process involves acid degumming, bleaching, and deodorization. The degumming of CPO was conducted in a degumming reactor, consisting of a heater, a mixture, and a centrifuge separator. The schematic diagram of the degumming reactor is shown in Figure 1. The degumming of CPO was conducted with varying time (15–30 min), phosphoric acid doses (0.03–0.06 wt%), and temperature (70–100 °C). The experiments were conducted based on the central composite design (CCD) of experiments, and the process was optimized using RSM. About 1000 g of CPO was taken in the reactor and heated up to 60 °C. Subsequently, 0.03–0.06 wt% of phosphoric acid was added into the reactor, and the mixture was agitated at 180 rpm, temperature 70–100 °C for 15–30 min. Then, the gum from the degummed oil was centrifuged at 12,000 rpm for 10 min. Then, the degummed oil was separated and bleached using bleaching earth at doses of 1.2 wt%, the temperature of 80 °C, and at a vacuum pressure of 60 mbar. Finally, the bleached palm oil (BPO) was deodorized under vacuum pressure of 6 mbar, temperature of 260 °C, and treatment time of 90 min. The refined bleached and deodorized (RBD) palm oil was collected for the analyses.

### 2.3. Design of Experiments of the Degumming Process

The impact of the degumming process on the mitigation of GE and 3-MCPDE in the RBD palm oil was determined by varying the degumming reaction time, phosphoric acid dosage, and temperature. The central composite design (CCD) was utilized to design the experiments of the degumming process of CPO. Then, the degumming process was optimized using RSM to obtain minimal GE and 3-MCPDE formation in the RBD palm oil. In the degumming process of CPO, the parameters of reaction time, phosphoric acid dosage, and temperature were independent variables, and the variables were coded based on the following equation [16]:

(1) $$X=\frac{x-\left[x_{max}+x_{min}\right]/2}{\left[x_{max}-x_{min}\right]/2}$$

where *X* is the coded variable, *x* is the natural variable, *x_max_* is the maximum level of the variables, and *x_min_* is the minimum level of the variables. The maximum, intermediate, and minimum level of the variables were coded as +1, 0, and −1, as shown in Table 2. The degumming process of CPO can be explained by the second-order polynomial equation, as shown in Equation (2) [16].

(2) $$Y=\beta_{o}+\overset{n}{\underset{i=1}{\sum }}\beta_{i}X_{i}+\overset{n}{\underset{i<j}{\sum }}\beta_{ij}X_{i}X_{j}+\overset{n}{\underset{i=1}{\sum }}\beta_{ii}X_{i}^{2}$$

where *Y* is the predicted yield of GE and 3-MCPDE in RBD palm oil, *n* is the number of coded variables, *β_o_* is the constant coefficient of intercept terms, *β_i_* is the constant coefficient of linear terms, *β_ij_* is the constant coefficient of interaction terms, and *β_ii_* is the constant coefficient of quadratic terms. The experimental data of the degumming process of CPO were analyzed and fit with the second-order polynomial equation using Design expert software (ver.11, Stat-Ease Inc., Minneapolis, MN, USA). The accuracy of the regression model was evaluated by an adjusted coefficient of determination (*R*^2^*adj*) and coefficient of determination (*R*^2^). The response surface plots were utilized to describe the interaction behavior in reducing GE and 3-MCPDE in palm oil.

### 2.4. Determination of the GE and 3-MCPDE in the RBD Palm Oil

The presence of GE and 3-MCPDE in RBD palm oil was determined following the Association of the Official Analytical Chemist (AOAC) Official Method Cd 29c-13 [17]. The analyses were conducted using Gas Chromatography (Agilent 7890B GC; Santa Clara, CA, USA) equipped with a Mass Spectroscopy detector (Agilent 6560 Ion Mobility LC/Q-TOF; Santa Clara, CA, USA) and Agilent capillary column HP-5MS (30 m × 0.32 mm × 25 μm). This determination of the 3-MCPDE and GE was conducted by following the indirect transesterification method. The transesterification method converts GE to 3-monobromopropanediol monoesters by the bromination process, which was followed by the acid transesterification to release 3-3-MCPDE from their esters phenylboronic acid derivatization. Finally, the quantification of GE and 3-MCPDE in RBD palm oil was conducted using gas chromatography with a mass spectrometry detector (GC–MS). The RBD palm oil (1.0 μL) was injected to the column in spitless mode at 250 °C. Helium was utilized as a carrier gas at a flow rate of 0.8 mL/min. The analysis was conducted by programming the oven temperature from 80 to 200 °C at 10 °C/min and holding the temperature for 10 min. The oven temperature was further increased to 300 °C at 15 °C/min and held for 15 min.

### 2.5. Analysis of RBD Palm Oil Quality

The RBD palm oil color was detected following the AOCS Official Method of Cc 13e-92 [17]. The RBD palm oil was melted at 60 °C, and a Lovibond tintometer Model F (Wilts, England) was used to detect the color. The color of oil was matched with standard colored and numbered glasses ranging in the scales from 0 to 70 red (R). The presence of FFAs in palm oil was evaluated following the titration method, according to AOCS Official Method Ca 5a-40 (AOCS 2009). The peroxide value (PV) in oil was determined by using the titration method, according to the AOCS Official Method Cd 8b-90. The oxidative stability of RBD oil was determined using the Rancimat method. This is an accelerated aging method to determine the oils and fats stability. The temperature was elevated at 110 °C, and rushing air was introduced during the analysis. Total chloride (TC) was analyzed using GC-MS following the AOCS Official Method Cd 29c-13 [17]. The phosphorus content in CPO and RBD palm oil was determined using the colorimetric method following the Malaysian Palm Oil Board (MPOB) test method of 2005 [18], which involved the ignition of the oil. The fatty acid compositions in CPO and RBD palm oil were determined using gas chromatography (GC), and the analyses of the fatty acids were performed by converting the fatty acids to their respective methyl ester (FAME). The FAME was analyzed using GC equipped with a flame-ionized detector (FID). About 0.1 μg of CPO and VPO were injected into the capillary column (100 m × 0.25 mm, id., 0.25 μm particles; Supleco, Bellefonte, PA, USA) with a split ratio of 1:10. The initial oven temperature was set to 40 °C, heated up to 100 °C at a 25 °C/min rate, and held for 25 min. Subsequently, the oven temperature further increased to 205 °C at a rate 10 °C/min and was held for 3 min. It was eventually increased the temperature to 240 °C at a rate 10 °C/min and was held for 10 min. Then, the temperature in the injector and detector was maintained at 250 °C throughout the analyses. Helium was used as a carrier gas.

## 3. Results and Discussion

### 3.1. Fitting the Regression Model

The degumming process parameters potentially influence the reduction of GE and 3-MCPDE formation in refined palm oil. Therefore, the optimization of the degumming process parameters is crucial to obtain the minimal concentration of GE and 3-MCPDE in RBD palm oil. The design of experiments was conducted based on the coded level of the three variables, including reaction time (*X*_1_), phosphoric acid doses (*X*_2_) and temperature (*X*_3_). A total of twenty simplified experimental runs were obtained, as shown in Table 3. The predicted yields for the GE (*Y_GE_*) and 3-MCPDE (*Y_3-MCPDE_*) concentrations in RBD palm oil were determined by applying the multiple regression analyses using the second-order polynomial equation shown in Equations (3) and (4), respectively.

(3) $$\begin{array}{ll} Y_{3-MCPDE}= & 3.190-0.03295X_{1}-2102X_{2}-0.0273X_{3}+0.000398X_{1}^{2}\\ & -10.1X_{2}^{2}+0.000092X_{3}^{2}+0.0056X_{1}X_{2}+0.000061X_{1}X_{3}\\ & +0.1833X_{2}X_{3} \end{array}$$

(4) $$Y_{GE}=4.944-0.08492X_{1}+33.05X_{2}-0.425X_{3}+0.000808X_{1}^{2}-242.2X_{2}^{2}+\;0.000143X_{3}^{2}-0.0583X_{1}X_{2}+0.000392X_{1}X_{3}-0.3194X_{2}X_{3}.$$

The predicted values obtained from Equations (3) and (4) for GE and 3-MCPDE concentration in RBD palm oil are presented in Table 3. A good agreement was found with the experimental values and predicted values for GE and 3-MCPDE concentration in RBD palm oil. Table 4 shows the estimated regression coefficient for 3-MCPDE and GE concentration in RBD palm oil. The intercept’s regression coefficient, linear, interaction, and quadratic terms were determined using least squire methods. In addition to the degree of significance and insignificance of studied variables, their interaction and quadratic effects were evaluated by assessing the *p*-values at the 95% (α = 0.05) of the confidence level [16]. It was found that the linear terms of reaction time and doses, the quadratic terms reaction time ∗ reaction time, and interaction between acid doses and temperature had a significant effect on the 3-MCPDE concentration in RBD palm oil. Conversely, linear terms of reaction time doses and temperature; the quadratic terms reaction time ∗ reaction time, acid doses ∗ acid doses, and temperature ∗ temperature; and interaction between reaction time and temperature, and acid doses and temperature had a significant effect on the GE concentration in RBD palm oil.

Table 5 shows the analysis of the variance (ANOVA) of the RSM quadratic model for GE and 3-MCPDE concentration in RBD palm oil. The correlation determination of the goodness of fit of the regression model was carried out by assessing the *R*^2^ and *R*^2^(*adj*) values [8,19]. Both *R*^2^ and *R*^2^(*adj*) values for the 3-MCPDE concentration in RBD palm oil were 0.9281 and 0.8755, respectively. The *R*^2^ and *R*^2^(*adj*) values for the GE concentration in RBD palm oil were 0.9915 and 0.9839, respectively. The *R*^2^ and *R*^2^(*adj*) values for GE and 3-MCPDE concentration in RBD palm oil were close to 1 and over 0.8 and 0.9, respectively, indicating a high degree of correlation between experimental and predicted values [7,20]. Moreover, the minimal pure error values and insignificant lack of fit values for GE and 3-MCPDE concentration showed that the regression model utilized was adequate for determining GE and 3-MCPDE concentration in RBD palm oil with the experimental range studied in the degumming process. The minimal residual values for 3-MCPDE and GE concentration indicate the good agreement between experimental data and the regression model utilized.

### 3.2. Analysis of the Response Surface

**Figure 2:**
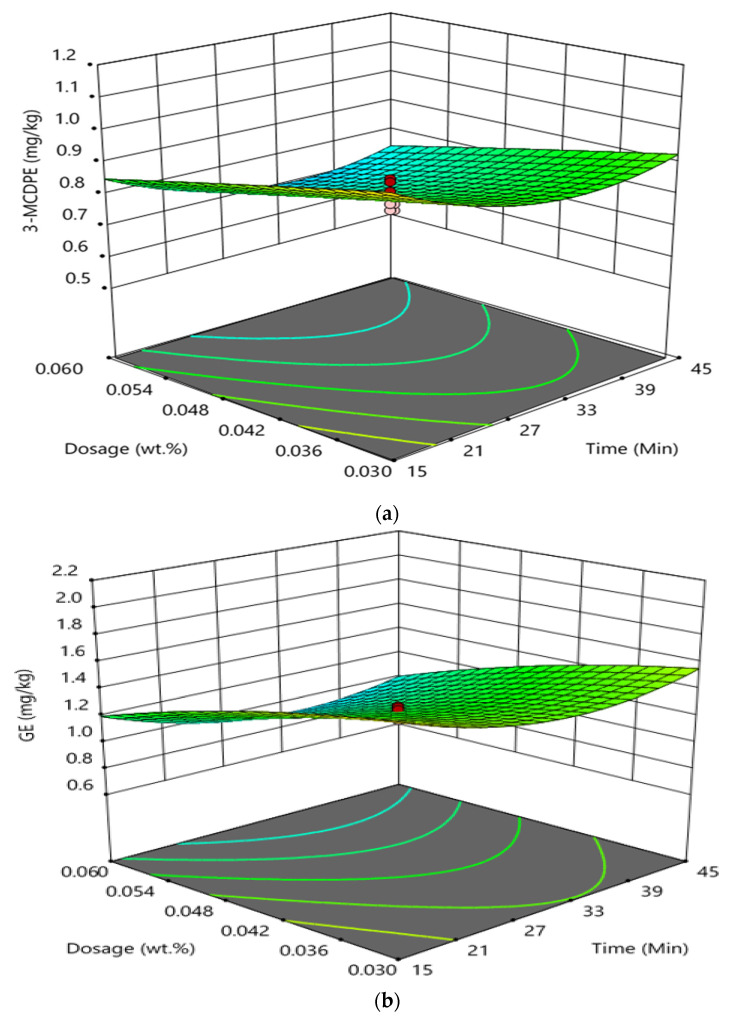
An interaction effect between reaction time and phosphoric acid doses in degumming process on the formation of 3-MCPDE (**a**) and GE (**b**) in RBD palm oil.

Figure 2 shows the interaction effect between reaction time and phosphoric acid doses of the degumming process of CPO on the formation of GE and 3-MCPDE in RBD palm oil. It was found that the interaction between reaction time and phosphoric acid doses of the degumming process had not significantly influenced the GE and 3-MCPDE formation in RBD palm oil. At a high reaction time and constant temperature of 85 °C, an increase in the phosphoric acid showed a negligible influence on the formation of the 3-MCPDE in the RBD palm oil. Wherein at high phosphoric acid doses and a constant temperature of 85 °C, the formation of 3-MCPDE decreased with increasing reaction time from 15 to 30 min and increased after that. The observation was found to be almost similar to the formation of GE in RBD palm oil. At a high reaction time and constant temperature of 85 °C, the increase in the phosphoric acid had a negligible effect on the formation of the GE in the RBD palm oil. Wherein at high phosphoric acid doses and a constant temperature of 85 °C, the GE concentration decreased with increasing reaction time from 15 to 30 min and increased with the further increase in reaction time.

**Figure 3:**
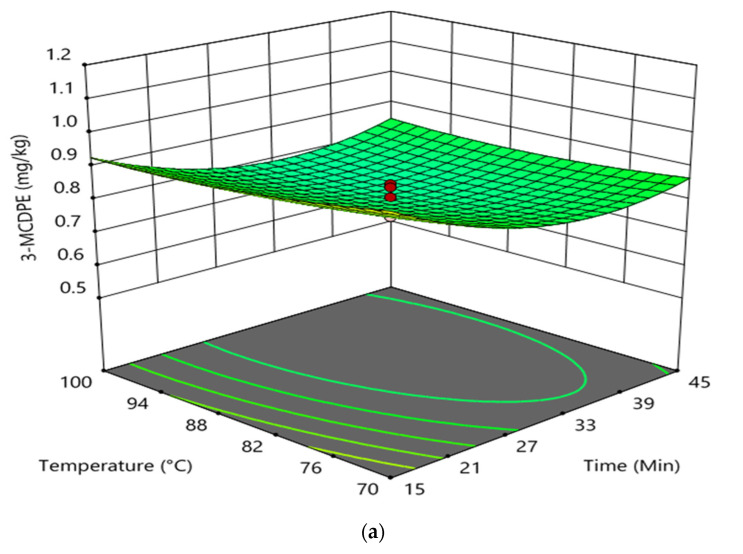

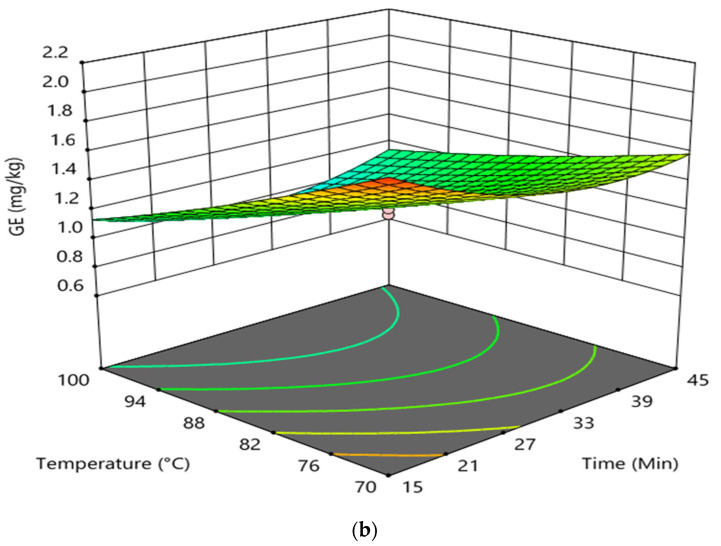
Interaction effect between reaction time and temperature in the degumming process on the formation of 3-MCPDE (**a**) and GE (**b**) in RBD palm oil.

The interaction effect between reaction time and temperature in the degumming process on the formation of GE and 3-MCPDE in RBD palm oil is shown in Figure 3. The interaction between reaction time and the temperature had an insignificant effect on the formation of 3-MCPDE in RBD palm oil. However, it had an insignificant impact on the GE formation in RBD palm oil. It was found that at higher reaction time and constant doses of phosphoric acid (0.045 wt%), the increase in temperature had a negligible effect on the 3-MCPDE formation in RBD palm oil. Conversely, the 3-MCPDE formation in RBD palm oil decreased with enhancing the reaction time up to 35 min and increased with an increase in reaction time at a higher temperature and a constant phosphoric acid dose of 0.045 wt%. In the case of GE formation at a higher temperature and at a constant dose of phosphoric acid (0.045 wt%), the increase in the reaction time slightly decreased the GE formation from 15 to 30 min and increased after that. However, the GE formation was sharply decreased with increasing temperature at higher reaction time and constant phosphoric acid doses. The elevated temperature of the degumming process increased the removal of gum present in CPO, which substantially prevents the GE formation in RBD palm oil and therefore decreased the GE formation with the increasing degumming temperature [8].

**Figure 4:**
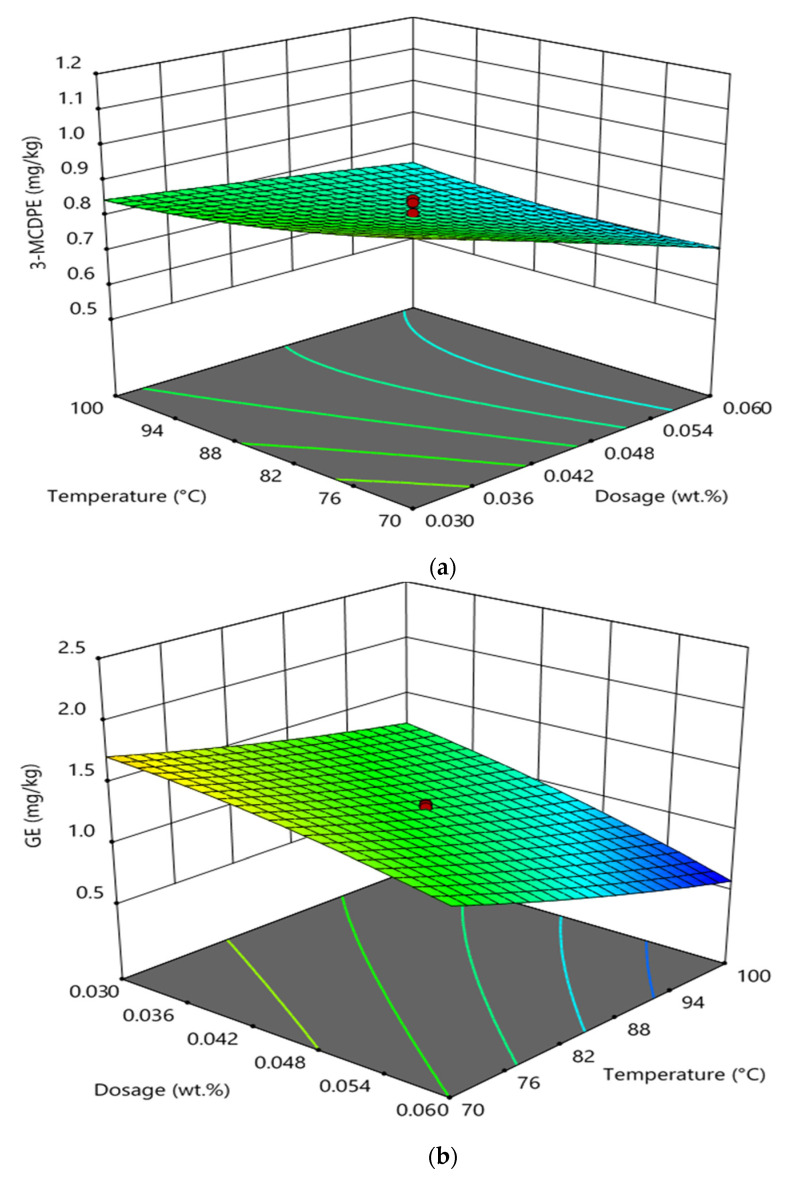
Interaction effect between temperature and phosphoric acid doses in the degumming process on the formation of 3-MCPDE (**a**) and GE (**b**) in RBD palm oil.

Figure 4 shows the interaction effect between temperature and phosphoric acid doses in the degumming process on GE and 3-MCPDE formation in RBD palm oil. It was observed that the interaction between temperature and phosphoric acid doses in the degumming process had a significant effect on the 3-MCPDE formation (Figure 4a) and GE formation (Figure 4b) in RBD palm oil. At higher phosphoric acid doses and constant reaction time (30 min) of the degumming process, the increase in the temperature from 70 to 100 °C decreased the 3-MCPDE formation in RBD palm oil. On the other hand, the increase in the phosphoric acid dosed from 0.03 wt% to 0.06 wt% decreased the formation of 3-MCPDE in RBD palm oil at higher temperatures and at constant reaction time (30 min). In the case of GE formation, the increase in the temperature from 70 to 100 °C sharply decreased the GE formation in RBD palm oil at a higher temperature and at constant reaction time. At higher phosphoric acid doses and a constant reaction time, the increase in temperature decreased the formation of GE in RBD palm oil [19,21].

### 3.3. Optimization of the Experimental Condition

The presence of gums in degummed palm oil causes the GE and 3-MCPDE formation during the deodorization process of palm oil due to the high deodorization temperature. Thus, the implementation of an effective degumming process is crucial to remove gums. It was found that the studied variables such as reaction time, phosphoric acid doses, and temperature of the degumming process and their interaction had influenced the GE and 3-MCPDE formation in the RBD palm oil. Hence, the optimization of the degumming process is crucial for reducing GE and 3-MCPDE formation in the RBD palm oil. However, the optimum experimental conditions of the degumming process were determined using Design Expert Software as follows: reaction time of 30 min, phosphoric acid concentration 0.06 wt%, and temperature 90 °C. Under this optimized experimental condition, the GE and 3-MCPDEformation in the RBD palm oil were determined to be 0.61 mg/kg and 0.59 mg/kg, respectively, which are below the recommended concentration by the European Food Safety Authority. The findings of the present study are comparable with studies conducted by Zulkurnain et al. [14] and Hew et al. [4]. Zulkurnain et al. [14] studied the influence of the physical refining process on the reduction of the 3-MCPDE formation in RBD palm oil. The minimal 3-MCPDE formation was reported to be 0.46 ± 0.05 mg/kg in RBD palm oil using a water degumming process, which was followed by bleaching with magnesium silicate and deodorization at 250 °C. Hew et al. [4] obtained the residual 3-MCPDE and GE in the RBD palm oil of 1.78 mg/kg and 0.31 mg/kg, respectively, using degumming of CPO with 0.06 wt% phosphoric acid, which was followed by the bleaching the degummed oil with 1.0 wt% BE and deodorization at 250 °C. With revising the refining process with the multiple degumming processes of CPO, the residual GE and 3-MCPDE concentrations in RBD palm oil were further reduced to 1.02 mg/mg and 0.16 mg/mg, respectively. In the present study, the residual GE and 3-MCPDE concentrations in RBD palm oil were found to be 0.61 mg/kg and 0.59 mg/kg, respectively. Wherein, the refined process involved acid degumming with 0.06 wt%, phosphoric acid at 90 °C for 30 min (optimal degumming process), which was followed by bleaching of the degummed oil using 1.2 wt% BE and deodorization of the bleached oil at 250 °C. Thus, it can be concluded that the phosphoric acid in the degumming process is a useful initiative to mitigate GE and 3-MCPDE formation in the RBD palm oil.

### 3.4. Determination of the RBD Palm Oil Quality

Table 6 shows the color, total chlorine (TC), free fatty acids (FFAs), peroxide value, phosphorus, and fatty acids content in CPO and RBD palm oil. The RBD palm oil was produced to determine the oil quality by treating CPO with the optimized phosphoric acid degumming process, which was followed by bleaching using BE at (doses of 1.2 wt%, the temperature of 80 °C, and at a vacuum pressure of 60 mbar) and deodorized the BPO under vacuum pressure of 6 mbar at 260 °C for 90 min. The phosphorus and FFAs content are the crucial properties to determine any vegetable oil quality. It was found that the phosphorus content in the RBD palm oil was reduced by 71.43% by refining the process. The residual phosphorus content in RBD palm oil was determined to be 1.50 mg/kg. Similarly, Zulkurnain et al. [14] found that the residual phosphorus content in RBD palm oil was 1.55 mg/kg. The phosphorus content reduction in RBD palm oil was due to removing hydratable and non-hydratable phospholipids in the degumming process. However, the phosphoric acid degumming process may donate the phosphorus content in degummed oil, and the BE effectively removed it during the bleaching process [21,22]. The FFAs content in CPO was determined to be 4.3 ± 0.51 wt%. However, the FFAs content was almost in nil in the RBD palm oil, revealing that the optimized degumming process potentially influences the removal of FFAs content in RBD palm oil in CPO refining. The residual color content in RBD palm oil was 1.9 ± 0.1 Red below the maximum value (≤3 Red) of refined palm oil color suggested by the Palm Oil Refiners Association of Malaysia [22]. The degumming process effectively removes the hydroperoxide content in RBD palm oil [23], which was assigned by reducing PV values in CPO and RBD palm oil (Table 6). The determination of the fatty acid compositions is vital in assessing palm oil’s nutritional value. Table 6 shows slight changes in the fatty acid compositions in CPO and RBD palm oil with the refining process. Palmitic acid was the most predominant fatty acid found in palm oil. The other major fatty acids found in the RBD palm oil were oleic acid and linoleic acid. In addition, the saturated and unsaturated content was almost 50:50. There is a slight increase in saturated fatty acid content and a slight decrease in unsaturated fatty acid content in RBD palm oil compared with the fatty acid compositions of CPO. This happened due to the breakdown of the unsaturated fatty acid chain during the degumming process [24].

## 4. Conclusions

In the present study, the phosphoric acid degumming process parameters of CPO were optimized using RSM based on the minimal formation of GE and 3-MCPDE in RBD palm oil. Optimal experimental conditions of the degumming process were determined as follows: reaction time of 30 min, phosphoric acid concentration 0.06 wt%, and temperature of 90 °C. Under this optimal experimental condition of the degumming process, the residual GE and 3-MCPDE concentration in RBD palm oil were 0.61 mg/kg and 0.59 mg/kg, respectively, below the European’s recommended concentration Food Safety Authority. Analyses of the adjusted regression coefficient (*R*^2^*adj*), the regression coefficient (*R*^2^), and insignificant lack of fit at 95% confidence reveals that the regression model was adequately fitted with the experimental data. The determination of RBD oil quality, treated with the optimum experimental condition of the degumming process, shows the effective removal of the phosphorus, total chlorine and hydroperoxide content, and FFAs. However, the phosphoric acid degumming process did not alter the properties of the fatty acids in RBD palm oil. Based on the finding of the present study, it can be postulated that phosphoric acid degumming is an effective process in the physical refining of CPO to remove phospholipids and FFAs with mitigating the formation of 3-MCPDE and GE in RBD palm oil and maintaining the nutritional value of the palm oil.

## Figures and Tables

**Table 1:** Characterization of the crude palm oil.

Properties	Unit	Amount
Free fatty acids	wt%	4.3 ± 0.51
Total chloride	wt%	3.39 ± 0.11
Peroxide value	meq/kg	2.0 ± 0.20
Deterioration of Bleachability Index (DOBI)	min	2.83 ± 0.14
Phosphorus	mg/kg	21 ± 2

**Table 2:** The coded and uncoded level of the independent variables.

Variable	Symbol	Level		
		Minimum (−1)	Intermediate (0)	Maximum (+1)
Reaction time (min)	*X*_1_	15	30	45
Phosphoric acid doses (wt%)	*X*_2_	0.03	0.045	0.06
Temperature (°C)	*X*_3_	70	85	100

**Table 3:** The central composite design of experiments and yields of 3-MCPDE and GE.

Run	*X*_1_	*X*_2_	*X*_3_	Yield (mg/kg)			
				3-MCDPE		GE	
				Actual	Predicted	Actual	Predicted
1	−1	−1	−1	1.16	1.14	2.02	2.07
2	1	−1	−1	1.01	0.99	1.73	1.74
3	−1	1	−1	0.89	0.87	1.67	1.71
4	1	1	−1	0.81	0.73	1.35	1.33
5	−1	−1	1	0.92	0.98	1.39	1.41
6	1	−1	1	0.89	0.90	1.47	1.44
7	−1	1	1	0.88	0.87	0.77	0.76
8	1	1	1	0.79	0.78	0.78	0.74
9	−1.682	0	0	1.16	1.15	1.96	1.90
10	1.682	0	0	0.93	0.96	1.56	1.61
11	0	−1.682	0	0.98	0.95	1.56	1.53
12	0	0	0	0.59	0.64	0.62	0.64
13	0	0	−1.682	0.83	0.90	1.90	1.86
14	0	0	1.682	0.87	0.82	0.77	0.80
15	0	0	0	0.85	0.80	1.17	1.24
16	0	0	0	0.84	0.80	1.26	1.24
17	0	0	0	0.75	0.80	1.28	1.24
18	0	0	0	0.77	0.80	1.27	1.24
19	0	0	0	0.80	0.80	1.25	1.24
20	0	0	0	0.81	0.80	1.21	1.24

**Table 4:** Regression coefficient and the significance of the quadratic model for the reduction of 3-MCPDE and GE in RBD palm oil.

Term	Coefficient		Standard Error		*T*-Value		*p*-Value	
	3-MCDPE	GE	3-MCDPE	GE	3-MCDPE	GE	3-MCPDE	GE
Constant	0.8027	1.2402	0.0225	0.0209	35.73	59.42	0.0001	0.0001
*X*_1_	−0.0540	−0.0866	0.0149	0.0138	−3.62	−6.25	0.005	0.0001
*X*_2_	−0.0927	−0.2648	0.0149	0.0138	−6.22	−19.12	0.0001	0.0001
*X*_3_	−0.0236	−0.3123	0.0149	0.0138	−1.59	−22.55	0.144	0.0001
*X*_1_^2^	0.0897	0.1818	0.0145	0.0135	6.18	13.49	0.0001	0.0001
*X*_2_^2^	−0.0023	−0.0545	0.0145	0.0135	−0.16	−4.04	0.879	0.002
*X*_3_^2^	0.0207	0.0321	0.0145	0.0135	1.43	2.38	0.184	0.038
*X*_1_ *X*_2_	0.0012	−0.0131	0.0195	0.0181	0.06	−0.73	0.950	0.485
*X*_1_ *X*_3_	0.0137	0.0881	0.0195	0.0181	0.71	4.87	0.496	0.0001
*X*_2_ *X*_3_	0.0412	−0.0719	0.0195	0.0181	2.12	−3.97	0.047	0.003

**Table 5:** Analysis of the variance (ANOVA) of the response surface quadratic model for 3-MCPDE and GE concentration in RBD palm oil.

Source	Degree of Freedom	Sum of Squares		Mean Square		*F*-Value		*p*-Value	
		3-MCDPE ^a^	GE ^b^	3-MCDPE	GE	3-MCDPE	GE	3-MCDPE	GE
Regression	9	0.2999	3.0604	0.0333	0.3395	10.99	129.65	0.0004	<0.0001
Residual	10	0.0303	0.0262	0.0030	0.0026				
Lack of Fit	5	0.0228	0.0174	0.0046	0.0035	3.03	1.98	0.1247	0.2363
Pure Error	5	0.0075	0.0088	0.0015	0.0018				
Total	19	0.3303	3.2012						

^a^*R*^2^ = 0.9281; ^a^ *R*^2^(*adj*) = 0.8755. ^b^ *R*^2^ = 0.9915; ^b^ *R*^2^(*adj*) = 0.9839.

**Table 6:** Quality and fatty acids properties of crude palm oil and RBD palm oil.

Properties	Unit	CPO	RBD Palm Oil
Color	Red	ND	1.9 ± 0.1
Free fatty acids	wt%	4.3 ± 0.51	0.01
Total chloride	wt%	3.39 ± 0.11	* ND (≤ 0.05)
Peroxide value	meq/kg	2.0 ± 0.2	ND (≤ 0.1)
Phosphorus	mg/kg	21 ± 2	1.5 ± 0.12
**Fatty acids**			
Dodecanoic acid (C12:0)	%	0.341 ± 0.01	0.204 ± 0.1
Myristic (C14:0)	%	1.085 ± 0.02	1.082 ± 0.01
Palmitic (C16:0)	%	43.486 ± 1.5	44.367 ± 1.4
Palmitoleic (C16:1)	%	0.118 ± 0.1	0.159 ± 0.1
Stearic (C18:0)	%	4.436 ± 0.10	4.376 ± 0.20
Oleic (C18:1)	%	40.220 ± 1.0	39.408 ± 1.2
Linoleic (C18:2)	%	9.391 ± 0.40	9.609 ± 0.35
Linolenic (C18:3)	%	0.272 ± 0.1	0.201 ± 0.2
Arachidic (C20:0)	%	0.476 ± 0.12	0.389 ± 0.10
Saturated	%	49.824 ± 0.5	50.419 ± 1.0
Monounsaturated	%	40.338 ± 1.0	39.567 ± 1.0
Polyunsaturated	%	9.663 ± 0.50	10.013 ± 0.40

* ND: Not detected.
